# Dataset of diatom abundances and diatom-inferred total phosphorus and electrical conductivity for Lake Tavatui (Middle Urals, Russi) over the last 11.7 cal ka BP

**DOI:** 10.1016/j.dib.2022.108300

**Published:** 2022-05-21

**Authors:** A.V. Maslennikova

**Affiliations:** Federal State Budgetary Institution of Science South Urals Research Center of Mineralogy and Geoecology, Urals Branch, Russian Academy of Sciences, Territory of the Ilmeny State Reserve, Miass, Chelyabinsk 456317, Russia

**Keywords:** Diatoms, Quantitative reconstruction, Lake sediment core, Urals, Total phosphorus, Electrical conductivity, Holocene

## Abstract

This paper presents the diatom data on Holocene sediments of Lake Tavatui (Middle Urals, Russia). The dataset includes abundances of 160 diatom taxa and quantitative reconstructions of total phosphorus and electrical conductivity of lake water through more than 11.7 cal ka BP. Diatom identification was performed using a Mikmed 6 var. 7 microscope, bright-field oil immersion optics at 1000 × magnification. At least 500 valves were counted to determine the relative abundances in the assemblages (diatom total, percentage). Quantitative reconstructions of lake water parameters were obtained based on the diatom inference models developed by simple weighted averaging and weighted averaging partial least squares methods. The data make it possible to refine the Middle Urals palaeoenvironmental reconstructions and to determine the main natural and human drivers on the Lake Tavatui ecosystem development during the Holocene. These data are an integral part of the original research paper (Maslennikova, 2022). Lake Tavatui sediment samples and diatom slide collections presented in this paper are kept in the South Urals Research Center of Mineralogy and Geoecology, Urals Branch, Russian Academy of Sciences (Russia). This dataset can be used in the regional comparison over the Holocene, in the research focused on human and natural impact on lake ecosystems, and in the investigation of diatom species distribution.

## Specifications Table

**Table:** 

Subject	Earth and Planetary Sciences
Specific subject area	Palaeolimnology
Type of data	Table
How the data were acquired	Counting was done with a Mikmed 6 var. 7 microscope using bright-field oil immersion optics at 1000 × magnification. Measurements for taxonomic identification were carried out with a ToupView 3.7. software, and photomicrographs were obtained with a ToupCAM UCMOS14000KPA digital camera. Transfer functions, reconstructions of electrical conductivity (EC) and total phosphorus (TP) were developed using a C2 software [2].
Data format	Raw data
Description of data collection	Lake sediment samples were treated with nitric and perchloric acids to remove organic matter. Slides were mounted using an Elyashev's mountant (*n* = 1.67–1.68) [3]. Diatom species on the slides were observed and identified with an optical microscope. Diatom species relative abundance data were log-transformed for the numerical analysis.
Data source location	Location of the Lake Tavatui sediment core: 57°13′05.85″, 60°17′98.89″. Diatom identification with the following numerical analysis were carried out in the South Urals Research Center of Mineralogy and Geoecology, Urals Branch, Russian Academy of Sciences (Miass, Russia).
Data accessibility	Repository name: PANGAEA Data identification number: https://doi.pangaea.de/10.1594/PANGAEA.943610
Related research paper	Maslennikova A.V. Holocene environments in the Middle Urals: Paleolimnological proxies from the Lake Tavatui (Russia). 2022. Quaternary International, 622: 51-64 https://doi.org/10.1016/j.quaint.2022.02.033

## Value of the Data

• The data will be useful for researchers who deal with Holocene palaeoenvironments, paleolimnology, diatoms, and people who are interested in the Urals environmental history.
• The data can be used for further research related to palaeoenvironmental reconstructions in the Urals
• This dataset can be applied in regional comparison over the Holocene for understanding of the local peculiarities of continental climate changes.
• The data are useful for comparative studies of lake ecosystem response to human and natural impact.

## 1 Data Description

The data are presented as diatom-inferred parameters of water (total phosphorus and electrical conductivity) during the last 11.7 cal ka BP and diatom abundances for 160 taxa in 73 samples of the Lake Tavatui sediment core (57°13′05.85″, 60°17′98.89″). In addition, the data include sample-specific standard errors (SEB) generated using 1000 bootstrap iterations for reconstructed water parameters. Diatom analysis was made for the composite sediment core of 3.5 m in length which was taken from Lake Tavatui (Urals, Russia) at 7.5 m water depth in 2009. The diatom nomenclature was updated using the Algaebase online catalogue [4]. Number of sample R407/1-75 includes the number of research carried out by the laboratory of mineralogy of technogenesis and geoecology (FSBIS South Urals Research Center of Mineralogy and Geoecology, UB RAS) and the number of Lake Tavatui sediment layer. All data are included into table (xls).

## 2 Experimental Design, Materials and Methods

For diatom analysis, samples were treated with nitric and perchloric acids to remove organic matter. Slides were mounted using an Elyashev's mountant (*n* = 1.67–1.68) [3]. Diatom identification was performed using a Mikmed 6 var. 7 microscope, bright-field oil immersion optics at 1000 × magnification. At least 500 valves were counted to determine the relative abundances in the assemblages (diatom total, percentage). Quantitative reconstructions of lake water parameters were obtained based on the diatom inference models developed by simple weighted averaging and weighted averaging partial least squares methods. For electrical conductivity reconstructions, a transfer function developed using a 72-lake regional diatom dataset was applied [5]. For total phosphorus reconstructions, a combined TP diatom dataset from the European Diatom Database Initiative (EDDI) [6–[1], [2], [3], [4], [5], [6], [7], [8], [9], [10], [11]] was used. Quantitative reconstructions were developed using a C2 software [2].

## Ethics Statements

No ethical guidelines or contents were relevant for the research reported on in this article.

## Funding

The quantitative reconstructions were supported by the Russian Science Foundation (Grant No. 21-17-00071, https://rscf.ru/project/21-17-00071/). The diatom abundance data were obtained with support of State Contract of South Urals Research Center of Mineralogy and Geoecology UB RAS.

## CRediT authorship contribution statement

**A.V. Maslennikova:** Conceptualization, Investigation, Writing – original draft, Writing – review & editing, Funding acquisition.

## Declaration of Competing Interest

The authors declare that they have no known competing financial interests or personal relationships that could have appeared to influence the work reported in this paper.

## Data Availability

Maslennikova, Anna Valer'evna (2022): Holocene diatom data from Lake Tavatui (Russia): diatom abundances and quantitative reconstructions of lake water parameters. PANGAEA, https://doi.pangaea.de/10.1 (Original data) (PANGAEA).

## Appendix Supplementary materials

Image, application 1
